# Challenges for the Mental Health of Refugee Artists: Perspectives of the ADAPT Model

**DOI:** 10.3390/ijerph20095694

**Published:** 2023-05-01

**Authors:** Halina Grzymała-Moszczyńska, Małgorzata Różańska-Mglej

**Affiliations:** 1 Department for the Psychology of Religion and Spirituality, Institute of Psychology, Jesuit University Ignatianum, 31-501 Kraków, Poland; 2 Faculty of Philosophy, Institute of Religious Studies, Jagiellonian University, 31-044 Kraków, Poland

**Keywords:** refugees, artists, mental health, ADAPT, IPA, ICORN

## Abstract

This study aimed to address the mental health challenges faced by refugee artists who are grantees of ICORN—the International Cities of Refuge Network—from the perspective of the extended conceptual ADAPT model. The study employed exploratory qualitative research, and data were collected through semi-structured interviews with ICORN artists in Poland, Norway, and Sweden. For data analysis, Interpretive Phenomenological Analysis (IPA) was used, whereas for the presentation of the results, the framework of the ADAPT model was applied. The results showed that the super-ordinate themes that emerged from the IPA analysis related directly to the ADAPT model and could mostly be assigned to its basic pillars: (1) Security; (2) Bonds and Networks; (3) Justice; (4) Roles and Identities; and (5) Existential Meaning. However, the model was insufficient for capturing the full diversity of experiences described by the respondents. Therefore, an extension of the model in the form of two additional pillars, Art and Body and Mind, was proposed. The findings confirm that the ADAPT model is adequate for systematizing and depicting in detail the experiences of migrants/refugees. However, further modifications of the model are necessary, particularly the additional pillar Body and Mind, which has the potential to become a separate category in other migrants’/refugees’ assessment frameworks. Moreover, Art itself could be seen as a universal bridging factor between the refugee and the host population, contributing to the refugees’ adaptation to the host society.

## 1. Introduction

The aim of this article is to address the mental health challenges of refugee artists who are grantees of ICORN—the International Cities of Refuge Network—from the perspective of the extended conceptual ADAPT model. This issue will be discussed based on the results of a qualitative study conducted with ICORN refugee artists between 2017 and 2022 in Poland, Norway, and Sweden. The main research question primarily encompasses factors that influence the acculturation process of the respondents once they reach a place of safe residence. Moreover, it also involves crucial elements of this process and the impact of these factors on the mental health of refugee artists from the perspective of the ADAPT model.

The respondents are grantees of the International Cities of Refuge Network, ICORN, which was launched in 2005 and now connects more than 75 cities around the world. The aim of the network is to provide long-term refuge for persecuted artists who often become targets of politically motivated harassment and attacks. The ICORN cities invite one persecuted artist at a time (alone or with family) and, in the form of a stipend, provide him/her with a place of safe residence and an opportunity to continue his/her work for a period of several years or longer. As of 2023, the network has hosted over 200 persecuted poets, novelists, playwrights, screenwriters, translators, bloggers, comic book writers, musicians, actors, publishers, and others [1].

All ICORN artists meet the refugee criteria as detailed in the Geneva Convention [2]; however, not all of them have legal refugee status. In host cities in Norway, for instance, artists automatically obtain refugee status and, consequently, are required to participate in a full integration programme. In contrast, artists residing in Sweden or Poland are only granted temporary residency.

### 1.1. Models of Perception of Refugees’ Mental Health

There are currently three perception models of refugees’ mental health, two of which focus mainly on dysfunction as a consequence of refugee trauma, whereas the other focuses on recovery.

The first and oldest model in question is the War Displacement Model, which posits that as a result of the multiple losses and traumas experienced by refugees in their countries of origin, they often suffer from PTSD once they reach their Western destination countries [3]. In this model, refugees are primarily diagnosed using the Harvard Trauma Questionnaire [4], which has been increasingly used to diagnose populations affected by war and other military conflicts [4].

A second, more contemporary model is the Ecological Displacement-Related Model, which, in contrast to the model discussed above, focuses mainly on refugees’ dysfunctions resulting not only from trauma experienced in the countries of origin but also in the countries of transit and/or destination [5]. It has been recognized that refugees’ mental health is also affected by, among other things, a dangerous migration path; prolonged living in poverty in overcrowded refugee camps [6]; unemployment; a loss of family, possessions, and social networks; and discrimination [3]. These factors have been shown to have at least as much if not more of an influence on the occurrence of PTSD symptoms among refugees than war experiences.

The third perception model of refugees’ mental health, which is discussed in this paper, is the ADAPT—Adaptation and Development After Persecution and Trauma [7]—model. It is a model developed by the Australian researcher Derek Silove and it focuses on demonstrating a wide range of dynamic psychosocial factors that can affect the mental health of populations experiencing mass conflict and displacement, including refugee populations [8]. It is a heuristic model that focuses on finding working solutions that can lead to recovery and is open to further analysis and development [7].

The ADAPT model is based on several key assumptions [7]:

(a) traumas experienced during conflicts have significant contexts, are simultaneous or sequential, and are complex in nature;
(b) there are several stages leading from trauma to psychopathology and at each stage, the individual can adapt positively, depending on their own resources. Therefore, deterministic models should not be used;
(c) there is a fluid and fine line between non-normative and normative psychological responses;
(d) the external world is a reflection of the individual’s internal world and the individual is in constant bilateral interaction with it;
(e) the healing process itself is an active process in which both individuals and societies naturally mobilize in order to adapt and survive;
(f) even in the most difficult conditions, post-traumatic growth and positive change are possible;
(g) in any society, including a post-conflict society, there is a certain percentage of the population that has symptoms of mental disorders both before and after the conflict.

ADAPT identifies five key psychosocial pillars seen as crucial to mental health and post-trauma recovery: (1) Security; (2) Bonds and Networks; (3) Justice; (4) Roles and Identities; and (5) Existential Meaning [7]. Pillar (1) Security refers to the sense of psychological and physical safety that is necessary for recovery and regeneration from trauma. Pillar (2) Bonds and Networks, in turn, encompasses interpersonal issues and a sense of being embedded in the world. Pillar (3) Justice usually refers to exposure to serious human rights violations. Pillar (4) Roles and Identities covers the loss of cohesion and sense of identity of individuals and whole communities [9]. The last pillar (5) Existential Meaning deals with changes in the area of faith and sense of meaning in life [10]. At the same time, the model also identifies psychosocial reactions that can damage or tear down each of the pillars at both the collective and individual levels [11].

### 1.2. Refugee Trauma

The first comprehensive definition of trauma appeared in the Diagnostic and Statistical Manual of Mental Disorders [12] (p. 236) and specified that trauma occurs when a person has to confront a potential threat to life or a dangerous event beyond their everyday life:

*The stressor producing this syndrome would evoke significant symptoms of distress in most people, and is generally outside such common experiences as bereavement, chronic illness, business losses, or martial conflict. The trauma may be experienced alone (rape or assault) or in the company of groups of people (military combat). Stressors producing this disorder include natural disasters (floods, earthquakes), accidental man-made disasters (car accidents with serious physical injury, airplane crushes, large fires), or deliberate man-made disasters (bombing, torture, death camps.)*

Over the years, this definition has been significantly modified by, among other things, the results of epidemiological studies on the frequency of exposure to trauma [13]. The subsequent definition of trauma provided in the Diagnostic and Statistical Manual of Mental Disorders from the year 2000 [14] was expanded to include the component of witnessing a traumatic event that befalls someone else and the subjective perception of the event in question as something overwhelming, causing fear, feelings of helplessness and terror. This means that an event can be traumatic for one person but not necessarily for another. The most recent definition of trauma can be found in the fifth edition of the Diagnostic and Statistical Manual of Mental Disorders from the year 2013 [15]. Although this definition retains the previously introduced condition of witnessing a traumatic event that affects someone else, it no longer includes a subjective perception component, as research has shown that an experience can be traumatic despite the lack of manifestation of emotions such as helplessness, fear, or terror [16].

Mention should also be made of the neurobiological determinants of trauma, which are, especially since 2000, an important area of research. Researchers have identified two distinct ways of coping with trauma: adaptive and non-adaptive [17]. The adaptive way assumes the individual’s ability to maintain healthy psychological and physical functioning while experiencing traumatic events [18]. The non-adaptive way of coping with trauma, on the other hand, assumes that trauma is located behind a wall that provides mental and emotional relief to the individual experiencing it [19]. The non-adaptive way of coping with trauma is particularly common among refugees, as migration forces them to adapt to life in a new country, redefine their family responsibilities, and focus on psychological strength. As a result, these refugees need to maintain the defence mechanisms that make this possible. However, if a non-adaptive way of coping with trauma persists, it can lead to chronic pathological reactions such as illness or criminal behaviour [20]. In the case of the non-adaptive way of coping with trauma, isolated memories of trauma persist and are constantly active causing a state of hyperactivity called ‘somatic memory’, which is one of the symptoms of post-traumatic stress. This state can persist as long as the traumatic memories have not been transformed and integrated [21].

Refugees who have fled their countries of origin for fear of persecution have often experienced or witnessed life-threatening situations, multiple losses, torture, murder, long-term stays in overcrowded camps, powerlessness, and suffering before or even during their journey [22]. Subsequently, they not only experience the stress of cultural adaptation in their new place of residence but also often feel treated as criminals [23]. Lengthy and complicated asylum procedures and continued uncertainty about their final legal status can further increase their stress levels [24].

As a consequence of the challenges they have experienced, refugees can experience normative and non-normative behavioural, emotional, and social reactions. Normative reactions include anger at the injustice experienced, an adequate response to danger, ensuring one’s own safety as well as that of their loved ones, or culturally accepted mourning. However, these reactions can also develop into non-normative reactions such as paranoia, hypersensitivity to signs of danger, prolonged grief, or depression [7].

Studies of mental health among refugees indicate a high rate of psychological disorders in this population compared to the other residents in their host countries [25,26]. The multiple traumatic experiences that refugees have faced in their countries of origin, during forced migration, and in their new places of residence can lead some to exhibit symptoms of refugee trauma, which is sometimes referred to as post-traumatic stress syndrome [22,27,28]. There can be many different symptoms of refugee trauma, including both somatic and psychological aspects [22]. These symptoms can be diagnosed and treated as a range of disorders, including psychosis, PTSD, and depressive and anxiety disorders, which are prevalent in this population [29].

### 1.3. The Role of Art in Coping with Trauma

As the group of respondents in this study consisted of artists working in the fields of literature, visual art, multimedia art, translation, comics, or music [30], examining the role of art in coping with refugee trauma was important.

To date, there have been relatively few publications on this issue. The available research primarily focuses on art therapy, including dance/movement therapy, movement and play therapy, BBAT, trauma-focused art therapy [20,31,32,33,34,35,36,37], arts activism, and expressive arts intervention [34,38,39,40,41].

Various studies have been conducted on both children and adults, including Syrian refugee children in Turkey [37], war-affected children from the developing world [32], youth detained at the Tornillo Influx Center in the USA [39], unaccompanied asylum-seeking children in Norway [34], and adolescent refugees from Burma in the USA [35]. Similarly, adult groups such as Chilean exiles in Europe, Canada, the United States, Latin America, and North Africa [38]; refugees residing at a nonprofit humanitarian organization in the USA [31]; traumatized refugees at the REFUGIO treatment centre in Germany [20]; traumatized refugees with PTSD in Denmark [33]; female refugees from Albania, Ghana, Iraqi Kurdistan, Iraq, Iran, Malawi, and Turkey in the UK [40]; refugees and asylum seekers with a PTSD diagnosis in Denmark [36]; and Syrian refugees resettled in Belgium [41] have been studied.

In terms of research methods, the most commonly utilized have been case studies/observations [20,31,32,41], surveys [34,35,36,37], and semi-structured interviews [33,38]. Deep and visual listening to art pieces [39] and participatory arts interventions [40] have been used less frequently.

The research findings confirm that including refugees in the creation of art is crucial both emotionally and existentially, i.e., it gives them hope that the future will be better [34,36,39], creates a coherent life narrative [32,38,41], bridges trauma and reduces social isolation [20,40], integrates experiences and emotions [33], reconstructs identities [41], and allows refugees to stand in solidarity with their compatriots who have remained in their home countries [38].

The groups studied in the research cited above are extremely diverse and heterogeneous and vary in terms of country of origin, age, and host country. The common element among them is that none of the respondents were professional artists but all of them were in the resettlement phase in their target host countries. However, no research was found on the role of art in coping with trauma among artists, especially among those who had been persecuted because of their artistic work. In addition, no research was found on the role of art in coping with refugee trauma in other stages of migration. The present study of ICORN respondents attempts to address the above-mentioned gaps, as it was conducted on persecuted artists and although it also covers the resettlement phase, it is heavily contextualized, as the artists surveyed have fresh memories of experiences from both the pre-migration and the transit phases.

## 2. Materials and Methods

### 2.1. Research Methodology

The study was conducted to gain an in-depth understanding of the experiences of refugee artists who are grantees of ICORN in Poland and Scandinavia in the area of coping with refugee trauma. To achieve the above research objective, exploratory qualitative research was conducted, as it allowed for a better understanding of poorly recognized phenomena and concepts [42] and enabled the study of specific phenomena within certain contexts [43]. Moreover, it can amplify the voice of ‘minorities’ [44] and be applied to the study of people from different cultures [45].

Data were collected through semi-structured interviews with ICORN artists in Poland, Norway, and Sweden, which were then transcribed. The aforementioned countries were chosen because most ICORN cities are located in Norway and Sweden, and conducting research in Poland was convenient for the researcher from a logistic and financial point of view. The respondents were asked about their experiences related to migration and adaptation, including their experiences with trauma, their perceptions of these experiences and the meanings they gave to them, the factors that helped them to cope with these experiences, whether art was helpful in dealing with refugee trauma and the role it plays in the host country, and the gains and losses associated with migration.

Because the starting point of the study was human experience, Interpretive Phenomenological Analysis (IPA) was used to analyze the data. IPA focuses on examining experiences that are meaningful to the respondents because they engage their thoughts and feelings while prompting them to reflect on the meaning of specific events in their lives. In recent years, IPA has been increasingly used in qualitative research, especially in the field of health sciences, social sciences, and psychology [46]. The method is also becoming increasingly popular in research involving migrants and/or refugees and/or those working with these populations daily [47,48,49,50,51,52,53,54].

To present the analyzed data in a clear and understandable way, the extended conceptual framework of the ADAPT model was applied.

### 2.2. Sample

Respondents were recruited partly through a personal network and partly through the ICORN Network Administration Centre, which is in regular contact with all former/current refugee artists. Individuals needed to meet two criteria for inclusion: they must have been an ICORN grantee for at least six months and have a proficient level of English communication.

The final group of respondents consisted of 18 ICORN grantees aged 31–63 who came from Iran, Iraq, Bangladesh, Egypt, Syria, Afghanistan, Zimbabwe, and Yemen. The fields in which the artists worked included poetry, visual art, prose, blog, comics, translation, journalism, multimedia art, performance, video art, music, acting, and arts management. Efforts were made to have an equal representation of men and women in the research group, but in the end, 14 men and 4 women were interviewed, reflecting the gender ratio among the ICORN grantees. The artists interviewed were all public figures who could easily be identified by the extremist groups or regimes that persecuted them in their home countries. Consequently, it is crucial to be careful when handling the data obtained from the artists and to anonymize their statements. Therefore, the respondents are referred to as Artist 1, Artist 2, Artist 3, etc.

### 2.3. Procedures of Data Analysis

As IPA provides both theoretical and practical guidelines for the analysis of the extracted data [46], the analysis procedures recommended by this method were applied. To organize and contextualize the large amount of data acquired, MAXQDA software was used.

In the first stage of the analysis, the transcript of a given interview was repeatedly and thoroughly read while the recording was being listened to. In parallel, the interviewed artists were given the transcripts of their interviews for review so that possible doubts could be clarified, errors made during the transcription could be corrected, and passages that may pose a threat to anonymization could be excluded.

The second stage, which was the most detailed and time-consuming, involved analyzing the content and language used in the text in question and writing down any comments or observations. During this process, the sections of the text that, in the researcher’s opinion, constituted separate units of meaning, were extracted.

In the next stage, the emergent themes were encoded, where the units of meaning extracted earlier were named. A given unit often had more than one meaning and was, therefore, also assigned to more than one code. According to IPA methodology, in this stage, the researcher coding the data is the central figure and the emergent themes are the result of a synergistic process of description and interpretation [46].

Stage four involved analyzing the relationships between the emergent themes and extracting the super-ordinate themes. These themes were then placed into theme tables [46].

Following IPA methodology, the analysis of each of the transcribed interviews consisted of the above-mentioned four steps, with the emergent themes and super-ordinate themes arising from the subsequent texts, thereby completing the tables of themes. The resulting tables were then compared with each other to extract the patterns present in all the narratives examined [46]. The group of respondents was quite large; therefore, it was important to identify and measure the repetition of the key super-ordinate themes by all respondents. Super-ordinate themes were defined as recurrent when they occurred among at least one-third of the respondents and covered at least 10 different text passages. The final set consisted of 28 super-ordinate themes and required the use of an appropriate ordering ‘key’ to better and more comprehensively understand the respondents’ experiences [55]. The best ‘key’ turned out to be the extended conceptual framework of the ADAPT model, allowing the list of super-ordinate themes to be ordered based on the five psychosocial pillars of ADAPT, i.e., (1) Safety and Security, (2) Bonds and Networks, (3) Justice, (4) Roles and Identities, and (5) Existential Meaning [7]. However, some identified super-ordinate themes could not be clearly assigned to the above five pillars and were, therefore, shown separately as additional pillars, that is, (6) Art and (7) Body and Mind. Subsequently, all the pillars and the super-ordinate themes that comprised them were ordered and presented according to their frequency of occurrence in the respondents’ narratives from most to least frequently occurring.

## 3. Results

The presentation of the survey results is based on the five super-ordinate themes that appeared most frequently in the statements of the artists surveyed: Community, Artistic Activity, Art and Migration, Art and Persecution, and Mind. A table of the most frequently occurring super-ordinate themes, together with their emergent themes and assigned to the respective ADAPT pillars, is provided below (Table 1).

### 3.1. Community

The super-ordinate theme assigned to ADAPT pillar (2) Bonds and Networks (see above) that occurred by far the most frequently (976 times) in the narratives of all 18 ICORN artists was that of Community. This theme comprises nine emergent themes, Community Back Home, Host Community, Family, Friends, Other Artists/Activists, Reference to Important Historical/Social/Social/Cultural Events/Persons, Differences/Similarities between Home and Host/Other Country, and Others, which are discussed in order from most to least frequently occurring and are shown in the table below (Table 2).

#### 3.1.1. Community Back Home

The emergent theme that occurred most frequently in the narratives of all respondents was the importance of community in their countries of origin. This theme evoked great emotion in the respondents and covered diverse aspects of their past and present experiences. Most respondents explained the circumstances of their migration and current events by focusing on the broad historical and social contexts of their places of origin; explained the current, often complicated geopolitical situations; and talked about the experiences and feelings of the inhabitants. Their communities, which often feel abandoned and alone, seem powerless and are dependent on other regional and global powers. Revolutions and social movements are important features of the communities back home. Some respondents experienced these phenomena as generators of change, whereas others felt that they had no transformative power. The theme of pervasive violence also emerged in all narratives. More often than not, this violence was deliberately used as a tool of oppression against unruly individuals such as the respondents themselves. All of them have been the victims of violence, whether physical or psychological, many have been threatened with death, and some survived assassination attempts. Sometimes, violence has been used against entire groups within a population. A good example of this can be seen in the narrative of Artist 10 (Iraq/Norway) who discusses the violence used by ISIS and al-Qaeda against Syrians in Mosul:

*Yes, after Saddam has gone in April 2003, Mosul was a very quiet and safe city until November 2004. I saw with my own eyes Daesh and Al-Qaeda coming: they have guns from Americans, in Ramadan they drink water, they’re smoking. They’re criminals. We know where they came from. From November 2004 until now—Mosul disappeared because of Islamists. They killed Syrian women—just because they were coming from Syria in 2004. I wrote some stories about that. One of them, she has been killed outside my house. She was lying there for 12 days. Because you cannot help them. If you call police, they will kill you and destroy your house. 12 days dogs were eating this body. Every day I saw this and I told my wife that maybe one day I’ll be like this. Outside my house (…)*

#### 3.1.2. Host Community

Another emergent theme comprising the super-ordinate theme of Community was Host Community, which was referred to by all respondents. The artists interviewed talked about their experiences in both the transit countries and the destination host countries. These stories featured both the violence and fear that are used as tools to manage migrants/refugees and the unequal opportunities migrants/refugees have when dealing with the systems of the transit/host countries. The communities in these countries often appear indifferent to their fate. The interviewed artists also described the challenges they and their families faced when fitting in with the host communities, including linguistic, educational, and work-related issues. Above all, respondents were concerned about their families fitting in with the new communities. The need to learn the language of the host country seems to be a particularly important issue for many respondents, as it allows them to interact on a deeper level with their local communities. Some of the artists were initially not motivated enough to learn, but over time, sometimes influenced by their children or other family members, they began to learn the language. Another strategy for deepening their interaction with the new community was to learn about its culture, i.e., the local mythology, and to look for parallels with the mythology of their countries of origin. Art practised by the respondents was also an important tool for interacting, as it allowed them to build a close and friendly relationship with the inhabitants of their host countries. Some of the respondents felt that the new country was full of opportunities, as long as their art was understood and respected, but at the same time complained about the lack of a network of close contacts that would support their artistic careers. Most of the respondents found their host communities to be quite open, helpful, and supportive in the context of their art, which made them keen to strengthen their interactions. Over time, the respondents can function in two cultures in parallel without any problems so they can also strongly influence the culture of the host country. However, they are aware that it is they who have to adapt to the local community and not the other way around. Furthermore, despite their integration, they experience the otherness and distinctiveness of the host country’s system in relation to the systems in comparison to those in their countries of origin. This awareness of separateness is perfectly described in the narrative of Artist 2 (Iraq/Norway):

*Oh my God, I’m going back again to that level and to that stage that I’ve been, I couldn’t write just because I’m surrounded with circumstances that I’m not in danger now, I’m safe but still the life is not easy to deal with and the system is killing me from inside. Even if it’s good system for me, for my benefit, for my kids’ benefit. But still you feel after while exhausted because your body, and your mind never adjusted to this system before, and you lived your life like a wildlife. And barely we have system to go through because everybody in Iraq may live like independent individual, and here they take care of everything. If I’m not to go to the hospital, I just go with my money. If I want to go to the doctor—I just pay, and I don’t wait for the system to come and help me.*

#### 3.1.3. Family

An emergent theme that occurred in most respondents’ narratives and also comprises the super-ordinate theme of Community is Family. Respondents regard their family as their responsibility, regardless of whether their relatives migrated with them or remained in their countries of origin. If the respondents migrated together with their families, the responsibility for them also emerges in the context of the journey itself and continues even after reaching the host country. The family itself is perceived ambivalently by the respondents, as on the one hand, they can count on help and support, but on the other hand, they often feel pressure from their families to change their life path. Because of this pressure, some artists decide to distance themselves from their families, whereas others prefer to rebel. Such was the story shared by Artist 6 (Egypt/Poland/Norway) who saw his family as a source of oppression:

*Since I was young, when I was at high school, I wanted to study medicine. When I didn’t get enough grades, I wanted to study something related. So, I went to study biology. But my parents weren’t happy with that, so they put a lot of pressure on me to change, they wanted me to study something related with the religion. So, I started something just because they wanted. But at the same time, I started my rebellion. I was being forced to do something that I didn’t want.*

#### 3.1.4. Friends

Another emergent theme discussed by most artists that comprises the super-ordinate theme of Community is that of Friends. The respondents often described their friends as an essential part of their everyday lives in their countries of origin. In most cases, friends were strongly supportive of respondents’ artistic activities and motivated them to continue their work, often being among the first recipients of their art. The respondents explained that in their countries of origin or transit, it was friends who supported them in moments of persecution, acted as their window to the world when they were in prisons or camps, and sometimes even saved their lives, despite often being persecuted themselves. Sometimes, it was friends who put the artists in contact with the ICORN network and motivated them to seek help. ICORN artists also described that on arrival in their host communities, their relationships with their friends were sometimes lost, and building new friendships in their host countries was difficult. The respondents coped with this by, e.g., staying in constant online contact with their friends in their countries of origin. However, some respondents such as Artist 11 (Egypt/Norway) mentioned the complete loss of previous friendships as a result of migration:

*The friends that helped me in Egypt, after I came here—they told bad things to me, they told that I’m coward. They told I run away as a rat. And one of them said: “Why you write about Egypt? You are not in Egypt, you’ve gone away.” They know my problems, they know what am I suffering, they know all things, but I don’t know why they do that. I cannot understand why.*

#### 3.1.5. Other Artists/Activists

Another emergent theme, which highlights another aspect of the discussed super-ordinate theme and was also mentioned by most respondents, is that of Other Artists/Activists. Within this theme, the artists described the different types of collaborations and networking, including both artistic and activist activities in local and international arenas. These collaborations took place before they migrated from their countries of origin, as well as in their host countries. The artistic community itself was enhanced by the respondents as they were competent and active, supporting and mentoring other artists on their development paths. At times, however, the community appeared opportunistic and focused on making a profit, which can have dangerous consequences, as it provides oppressive governments with the opportunity to persecute entire groups of artists/activists who are critical of them. Although most of the statements made by the artists interviewed involved the artistic/activist community in their countries of origin, they also share reflections on this community in their host countries. In particular, they mention that they miss such a community and are constantly searching for it or trying to create it. Among others, Artist 10 (Iraq/Norway) talks about such an experience in his narrative:

*Yes, bigger city—to find someone who understands me, to find a group having the same idea, working together. In Norway you cannot find. In Tromso I had one friend. I must travel eleven hours.*

#### 3.1.6. Reference to Important Historical/Sociopolitical/Social/Cultural Events/Persons

Another theme that comprises the super-ordinate theme of Community and was addressed by most respondents is that of Reference to Important Historical/Sociopolitical/Social/Cultural Events/Persons. The presence of this theme shows that the artists interviewed have in-depth knowledge and can reflect on both their countries and cultures of origin and other regions of the world. In their narratives, they addressed historical events such as wars and revolutions, which have had a major impact on the trajectories of their lives and those of their loved ones, as well as contemporary issues in the transit and host countries. Respondents seemed knowledgeable about the socio-political situation in the regions to which they migrated and tried to apply the positive solutions they learned there to their communities in their countries of origin. In their statements, respondents often referred to historical figures, not only from their cultures of origin but also from other cultures. An example of this can be seen in the statement of Artist 9 (Egypt/Norway) who refers to the figure of De Gaulle:

*Not “I am France” that means “I lead France”. De Gaulle meant: “I’m the leader”. “I am Sinai” but not leader, I feel as something from Sinai as trees, all details.*

#### 3.1.7. Differences/Similarities between Home and Host/Other Country

Another emergent theme that occurred frequently in the narratives of most ICORN artists is that of Differences/Similarities between Home and Host/Other Country. The similarities mentioned by the respondents related primarily to ideological issues. The respondents perceived certain topics as universal, occurring not only in their countries of origin but also in other countries. In the case of the ICORN artists, these similarities may be fairly obvious, for example, the geographical features of the origin and host cities or the rhythm of everyday life. However, they may also be quite surprising. Some respondents stated, for example, that for various reasons, in both their country of origin and host country they were not able to continue their artistic work. Moreover, in both places, they were perceived as rebels and had equally difficult financial situations. In contrast, the differences experienced by the respondents, for example, the feelings of security and freedom or interest in their work, were often in favour of the host countries. However, this was not always the case, as some artists felt that their everyday lives had deteriorated in their host countries, especially in terms of opportunities for artistic development. Artist 8 (Syria/Norway) spoke of such a situation:

*Some writers wrote a lot but they didn’t get chance to publish anything. So, what am I doing here? If I was in my country, and writing all these things—I would publish them. It’s because of the difference in culture, and the traditions, and the interests of people.*

#### 3.1.8. Others

The final emergent theme that comprised the super-ordinate theme of Community is that of Others. Others refers to the various non-artistic people the respondents have met throughout their lives. Typically, these are ordinary people who lead ordinary, predictable lives. The respondents mainly mentioned people they had met in the context of their artistic activity while still in their countries of origin. Others they had encountered during migration, for example, in transit countries, are seen as potential sources of help and hope for improved support and outcomes, as exemplified by the experience of Artist 17 (Zimbabwe/Sweden):

*At the same time I had hope from the discussion I had with the fellow people in South Africa. There’s an Indian guy with whom I was staying with. He actually gave me a shelter. I felt I just need to die. By sharing this situations, I pulled my heart out, I cried—then at times I felt I’m getting better.*

### 3.2. Artistic Activity

The second super-ordinate theme discussed here is that of Artistic Activity, which occurred 373 times in the narratives of all 18 ICORN artists surveyed. This theme belongs to the additional ADAPT pillar, Art. Artistic Activity consists of six emergent themes, Artistic/Activist Path, Audiences, Genres of Art Practiced, Current Artistic Activity/Activism, Being a Famous Artist/Activist/Publisher/Translator, and Inspiration / Motivation, which are discussed below in order from most to least frequently mentioned and are presented in the table below (Table 3).

#### 3.2.1. Artistic/Activist Path

The first emergent theme that comprises this super-ordinate theme is that of Artistic/Activist Path, which was present in the narratives of all the artists interviewed. Within this theme, many of the respondents talked about the diverse beginnings of their artistic paths and shared their reflections on whether art was their primary passion or a career path. Their development paths appear to have been determined by local circumstances and opportunities such as a lack of art education, funding, or materials. Many respondents described themselves as self-taught artists who became artists against the odds as a result of their own work and perseverance. Respondents also shared memories regarding their artistic debut and key interactions with other artists. An issue raised by some of the respondents was the diversity and versatility of their art and its combination with activism. For many of them, journalism was the path that led them to activism and allowed them to embed their art in a broad socio-political context. Such an experience was shared, among others, by Artist 14 (Iran/Norway):

*Does this kind of life change my poetry and literature as well? I didn’t decide to use literature or poetry as a tool for my activism. But it happened. When your life changes, your mind changes—your poetry changes.*

#### 3.2.2. Genres of Art Practiced

The second most common emergent theme is that of Genres of Art Practiced, which was addressed in the narratives of all 18 artists interviewed. Within this theme, many respondents emphasized that they moved freely among several art genres. Some, for example, combined traditional disciplines such as journalism, prose, and poetry, or television, radio, and theatre, whereas others worked within new genres such as multimedia performance or blogs. Interestingly, respondents were also often active in areas that accompany the arts such as working as publishers or translators. However, not all respondents were versatile and some of them deliberately limited their activities to one art genre so as not to become distracted. Some artists also shared critical reflections on different types of artistic activity. A good example of this is Artist 2 (Iraq/Norway) who spoke critically about journalism, which she believes kills all creativity:

*Most popular job for writers is journalism. And after a while you’ll hate writing because of journalism, sometimes it kills the creative part of you because it’s not the creative thing, it’s not fictional, it’s reality, you’re dealing with reality. But you have to continue to not lose your name and so the people don’t forget you. So this kind of things affect our careers as artists, as fictional writer, and after a while you become like a machine repeating the same thing just because it’s the political market’s demand, if you understand what I mean. The situation demands for that. This upsets me, personally—most of my career.*

#### 3.2.3. Audiences

Another emergent theme that comprises the super-ordinate theme of Artistic Activity is that of Audiences, which was present in the narratives of most ICORN artists. Some respondents mentioned that sometimes their first audiences were friends and family but most focused their stories on the differences between audiences in their host countries and those in their countries of origin. The respondents stated that their audiences in their countries of origin seemed engaged, numerous, and had a deep understanding of their art, but after migration, the respondents no longer observed these characteristics in their audiences. Consequently, in order to exist in front of audiences in the host country, respondents have had to adapt to the locally prevailing rules of the game and existing structures. Respondents within this core theme also talked about their specific relationship with their audience. Some wish to create in a way that can be understood by any audience, regardless of their country of origin, whereas others see the audience as an essential part of the creative process, providing a driving force and motivation to continue creating. Artist 8 (Syria/Norway) talks about such a situation in his narrative:

*And the readers there in the prison encouraged me a lot—especially when I began with translating the “Blindness”—Saramango’s novel. When I began translating it, they began reading it chapter by chapter. Whenever I finished a chapter, they take it and read it and urged me to continue translating because they wanted to read.*

#### 3.2.4. Current Artistic Activity/Activism

Another emergent theme that appeared in the narratives of most of the artists interviewed was that of Current Artistic Activity/Activism. Many of the respondents talked about the fact that in terms of their artistic position in their host countries, they often had to build it from scratch, on top of having additional activities and responsibilities due to migration. Respondents often tried to become even more versatile in terms of the art genres they deal with in order to increase their chances of establishing themselves in the local environment. Their artistic activity seems to be a bridging factor between them and the host population, thereby contributing to their adaptation to local society.

A large proportion of ICORN artists continued to be active in their communities of origin in both artistic and activist forms while living in their host countries. At the same time, the respondents were also often active in their host communities by collaborating with local artists, writing in foreign languages, translating texts from the host country’s language into their native language, or volunteering in local projects. The majority of respondents also emphasized that the fact that they could continue their artistic/activist activities while migrating was important to them. This was recounted in the narrative of, among others, Artist 2 (Iraq/Norway) who was already planning her next novel while still working on the previous one:

*But all the time I think ok—I’m finishing the novel I’m working on, I’m done 80% of it, and after that I ask myself—ok, you’re done with the most of the city but what else? What’s coming? So, every time, each time I think about something else. I don’t know what will come next, but still asking is something that brings hope. We will begin again. I will begin again. So, I will not stop.*

#### 3.2.5. Being a Famous Artist/Activist/Publisher/Translator

Another emergent theme that comprises the super-ordinate theme of Artistic Activity and occurred in most of the narratives of the interviewed artists was that of Being a Famous Artist/Activist/Publisher/Translator. Quite a number of respondents shared their experiences of being famous and popular in the countries from which they emigrated. Often, their recognition not only served themselves but also the larger cause for which they fought. However, many respondents experienced a loss of their status and fame as a consequence of migration, especially those who were primarily known in their respective cultural circles. Respondents who were known to a wider international audience before migration did not experience this loss upon arrival in their host countries. In contrast, respondents who were not yet well-known and popular before migration hoped to become famous in the future. This is exactly the plan that Artist 5 (Iran/Norway) mentioned in his narrative:

*No, I have ideas about the next step. I want to be famous, I’ll do a lot of things, I’ll start film-making. I’m going to be very famous, ok?*

#### 3.2.6. Inspiration/Motivation

The final emergent theme within this super-ordinate theme is that of Inspiration/Motivation, which was present in the narratives of some of the artists interviewed. Some respondents stated that mostly people inspired their work, including their audiences, loved ones, and the local community. For some, an inner conflict or the challenges and problems experienced in their communities of origin can also be a motivation. In contrast, socially relevant issues in their host countries were described by them as either too trivial and ordinary or too heavy to be inspired by. However, some of the respondents, including Artist 7 (Egypt/Sweden), believed that even the most difficult experience can be inspiring for an artist, provided the right perspective is gained over time:

*Everything can be helpful for a writer. Every experience—to be humiliated in your daily life is a very good thing I think. It can support your writing. But I don’t feel the effect now—maybe in the future I will get it, like a reward. But now—no, I can’t see it from this close position. The perspective is too short. In this case—maybe after I’ll be here for a couple of years.*

### 3.3. Art and Migration

The third super-ordinate theme discussed here, which was assigned to the additional ADAPT pillar, Art, and appeared 246 times in the narratives of 17 ICORN artists, is the theme of Art and Migration. This theme consists of five emergent themes, Migration and Being an Artist, Choice of Migration, Stopping Creation in Exile, Continuing Creation in Exile, and Disadvantages of Migrating as an Artist, which are presented below in order from most to least often discussed and are shown in the table below (Table 4).

#### 3.3.1. Migration and Being an Artist

The theme that occurred most frequently in the artists’ narratives was that of Migration and Being an Artist. The vast majority of respondents never wanted to migrate or live in exile, but persecution due to their artistic and activist work forced them to do so. ICORN artists also discussed the challenges they faced when migrating as artists. They mentioned, among other things, the changes in the form and content of their art, the difficulties of entering a new artistic environment, the different artistic sensibility of the local community, the loss of social motivation for their work, and the lack of opportunities to work in their profession. Artist 13 (Afghanistan/Sweden), among others, talked about this issue:

*I talk with some friends and they show some options, but until now I’m a little worried because in these three months I met some Swedish journalists, I visited some newspapers’ offices like “Sydsvenska Dagbladet” and also the radio in Malmö. I couldn’t find any journalists from other countries. All of them are Swedish. Because of this, I think it’s hard for me to work as a journalist here.*

#### 3.3.2. Choice of Migration

Another emergent theme comprising the super-ordinate theme of Art and Migration, which was discussed by most of the artists interviewed, is that of the Choice of Migration. Some of the artists interviewed view migration not as their own choice but as something that happened to them. They felt that it was a particular situation that forced them to leave and that they did not fully consent to leaving. Other respondents, on the other hand, felt that migration was an autonomous choice but that the country they migrated to was not necessarily chosen by them. Such an experience was shared by, among others, Artist 2 (Iraq/Norway) for whom the choice of Norway as a host country was simply the best option due to the fact that she was travelling with her children:

*No, it was the best choice available for me as a family because if you chose to go to Sweden for example, it was risky not to get permanent residence. But here you can live as a refugee and pend residency. Because I wasn’t alone, I can move with my two kids.*

#### 3.3.3. Stopping Creation in Exile

The third emergent theme in the narratives of the artists interviewed that comprises the super-ordinate theme of Art and Migration is that of Stopping Creation in Exile. The respondents shared the reasons they had to stop creating in their host countries, including a lack of time due to additional work or the objective impossibility of continuing their work, for example, as an investigative journalist. Some respondents also told us that they were unable to continue creating due to mental or psychological problems. Another reason for stopping their artistic/activist activities was the need to protect their families, who have stayed in their home countries and could suffer if the respondents continue their work. This emergent theme also includes general reflections by the respondents on the reasons for ceasing to create in exile, including the inability to use the regained freedom of expression or the temporary loss of contact with one’s art, as described by Artist 2 (Iraq/Norway):

*But this stopping thing, I think it’s very common for writers, they stop for a while because they lose the connection with the work, with the figures, with every detail, but suddenly you take them back again. We don’t know how. It’s just like that. Something happens.*

#### 3.3.4. Continuing Creation in Exile

Another emergent theme addressed by some of the ICORN artists was that of Continuing Creation in Exile, a theme that contradicts the emergent theme discussed above. Some of the respondents stated that they were very active in their host countries and that migration had not changed anything in this regard. The respondents often continued to work on challenging and socially engaged topics related to their countries of origin. Many respondents also stated that they needed to keep creating in order not to turn into ordinary non-artistic people over time. An important concern that respondents also shared was the migrant-artist trap that ICORN artists often fall into in their host countries. A good illustration of this issue was the rhetorical question asked by Artist 14 (Iran/Norway):

*I published my books, I participated in gatherings, conferences but how many times you can ask me or call me a writer in exile?*

#### 3.3.5. Disadvantages of Migrating as an Artist

The final emergent theme addressed within the super-ordinate theme of Art and Migration by some of the artists interviewed was that of the Disadvantages of Migrating as an Artist. A key issue raised by the respondents was the experience of multiple losses, which include the loss of access to one’s community, professional work, personal life, education, and previous standard of living. In addition, respondents stated that by migrating as artists, they had lost the opportunity to publish, their audience and the feeling of being understood by them, and sometimes even the opportunity to create in a particular genre. Many respondents, including Artist 4 (Bangladesh/Norway), felt that migration was associated with more challenges and problems for artists than for other people:

*But when it comes to an artist, a writer—I think he’s facing more problems, some other reality. As a creative person always his thinking, his lifestyle—everything is something different. When he left all of his chances and others, he becomes mad.*

### 3.4. Art and Persecution

The last super-ordinate theme within the ADAPT pillar Art, which occurred 214 times in the narratives of all artists interviewed, was the theme of Art and Persecution. This theme consists of five emergent themes, Persecution in the Home Country, Protection Strategies, Story of Persecution, Possibility/Impossibility of Going Back to the Home Country, and Censorship/Self-Censorship, which are discussed in order from most to least frequently mentioned and are depicted in the table below (Table 5).

#### 3.4.1. Persecution in Home Country

Persecution in Home Country was the emergent theme most often mentioned by respondents. A form of persecution frequently mentioned by the artists interviewed was imprisonment, which was a potential consequence of their work. Most of the respondents were fully aware that their work could cause a harsh reaction from those around them and that, as a consequence, they could be persecuted. Other common forms of persecution mentioned were constant surveillance, a total ban on publication, preparation of evidence against them, or frequent arrests. Interestingly, arrest was sometimes viewed by respondents as a lesser evil, as it enabled them to survive compared to staying at large, which could expose them to physical attacks or even murder attempts. This was the experience shared by Artist 13 (Afghanistan/Sweden):

*Yes. In 2014 when I work in Mazar-i-Sharif, it’s the city in North Afghanistan, one night when I left the office, I was on the way walking across the road, and 2 gunmen came and attacked me. It was because before I had published some article about warlords and election, provincial election and also fundamentalist groups. They told me: “Why you write something about me, about us?”. I asked them: “Who are you? Are you terrorist, are you my enemy? I don’t have any enemy. Why?” They attacked me. Maybe it’s fine now. But they attacked me with a pistol and some knife.*

#### 3.4.2. Protection Strategies

Another emergent theme that comprises the super-ordinate theme of Art and Persecution is that of Protection Strategies. A protection strategy commonly used by respondents was hiding and fleeing to another region or country. The respondents tried to change their place of residence and work frequently so that it would be harder to trace them. Another protective strategy frequently used by respondents was the use of secure instant messaging services such as WhatsApp. A reverse strategy, the use of which is mentioned in the narrative of Artist 14 (Iran/Norway), is that of transparency:

*I thought that I didn’t have anything to hide. Really, I was living in a glass home. They controlled me everywhere, and I don’t want to hide something. Everything I found I published and I was talking about it. Even in prison or under investigation. I thought that this is the best protection.*

#### 3.4.3. Story of Persecution

Another emergent theme that occurred frequently in the narratives of ICORN artists was that of the Story of Persecution. The respondents made it clear that persecution was caused by their artistic/activist activities. The descriptions of the individual stories of persecution were extensive and extremely detailed and often included both an explanation of the political/social background and a recall of violent reactions to the respondents’ actions. As part of this emergent theme, respondents also talked about their exit strategies from situations of persecution, including leaving their countries of origin within the framework of the ICORN scholarship. Sometimes, however, contrary to their expectations, the story of their persecution has no end, and they continue to be harassed and persecuted remotely after migrating, which happened to Artist 16 (Bangladesh/Sweden):

*They’re observing my social media—my Facebook account is so much vulnerable nowadays. Someone informed me that you just deactivate your account, otherwise they’ll disable your account. It happened before 4 or 5 times they disabled my ID. I deactivated my account for 7 days. Now I have opened. But every time I’m in stress that now they’ll hack my Facebook. They can do anything. (…) They’re monitoring me—what I’m writing in social media.*

#### 3.4.4. Possibility/Impossibility of Going Back to Home Country

Another emergent theme that comprises the super-ordinate theme of Art and Persecution is that of the Possibility/Impossibility of Going Back to the Home Country, which was raised by most of the respondents. Within this theme, the respondents shared their desire to return to their countries of origin, but unfortunately, it is not feasible in many cases because the local situation does not allow it. In these cases, a return would only be possible if the political and social situation changed considerably. In addition, respondents also mentioned another obstacle to returning to their countries of origin, namely that their families had already integrated into the local communities in the host countries to the extent that leaving would be impossible. Artist 3 (Bangladesh/Norway) mentioned such an experience in her story:

*Yes, the first thing—if I want to go back to my own country; even though things have changed in Bangladesh—there’s very little opportunity. But my son and daughter, they’re getting Norwegian education and particularly my daughter, she was only 8, so she has almost forgotten reading in Bengali. So, if she went back she would not be able to communicate. That’s one part.*

#### 3.4.5. Censorship/Self-Censorship

The last emergent theme that was discussed by half of the respondents comprises the current super-ordinate theme is that of Censorship/Self-censorship. The censorship discussed by the artists was primarily connected with their work and was imposed by the state apparatuses in their countries of origin. This censorship was enforced by both state institutions and individuals such as magazine editors. However, many respondents also mentioned self-censorship, which they imposed on themselves in order to avoid possible attacks as a result of publishing banned content. Artists also mentioned an aspect of the censorship they experienced in their host countries, namely ostracism. Artist 6 (Egypt/Poland/Norway) shared such an experience:

*And also, I found out that censorship is international thing. The only difference is that the consequence of expressing yourself are different in other places, but you still have consequences. Here in Norway, just like being avoided, you are not welcomed in some circles, they don’t like you. You might be kicked out.*

### 3.5. Mind

Last but not least is the super-ordinate theme of Mind, which occurred 241 times in the narratives of the artists interviewed and belongs to the additional ADAPT pillar, Mind. It consists of four emergent themes, which are shown in the table below (Table 6) and discussed from most to least frequently occurring, Emotions as an Artistic Migrant, General Emotions in Migration, Fear, and Emotions during Persecution.

#### 3.5.1. Emotions as an Artistic Migrant

The emergent theme that occurred most frequently in the narratives of all respondents was that of Emotions as an Artistic Migrant. Respondents talked about difficult emotions related to their migration, including a feeling of rejection of their work by both their host and home communities, sadness connected with forced migration, a sense of not being connected to the local art community, or a sense of not being welcome as an artist. However, respondents also experienced positive emotions, which included the joy of being an artist in their new place of residence and the feeling of happiness when the host community understood and appreciated the art they created. This feeling of joy in the host country was experienced by Artist 15 (Iran/Norway):

*He was happy that someone else, from another language, of totally different background actually understands, and approves and gets really interested in his poems.*

#### 3.5.2. General Emotions in Migration

The next emergent theme comprising the super-ordinate theme of Mind that occurred in the narratives of most of the artists interviewed is that of General Emotions in Migration. The emotions that the respondents felt when they emigrated ranged from feelings of happiness at being in the host country and being able to do what they wanted to feelings of longing for their countries of origin or depression when finally reaching their host countries. Artist 5 (Iran/Norway), among others, talked about the difficult emotions experienced during the first weeks of the ICORN scholarship:

*I was here for 2 weeks, the next 2 weeks I was in a total crisis. I was so depressed. I used to forget eating, I still do—I’m waiting for someone to call me to eat as they did in the compound. Then it got better.*

#### 3.5.3. Fear

The emergent theme frequently raised by most ICORN respondents in the context of the discussed super-ordinate theme is that of Fear. Fear, as described by respondents, constantly accompanied both them and other artists in their countries of origin. For some respondents, this fear subsided upon arrival in their host countries, but for others, it persisted and accompanies them daily. This is the case for Artist 14 (Iran/Norway) who stated:

*Honestly, I’m scared every single day. It’s not finished.*

#### 3.5.4. Emotions during Persecution

The final emergent theme that comprises the super-ordinate theme of Mind is that of Emotions during Persecution. The emotions mentioned in the context of persecution by most respondents were stress and horror, although a sense of indifference to one’s fate and future was also evident in the narratives. However, a number of artists, including Artist 18 (Yemen/Sweden), also mentioned resilience and the idea of not giving up in the face of persecution:

*But then I didn’t give up. I try to live.*

## 4. Discussion

The results of this study showed that the super-ordinate themes that emerged from the IPA analysis related directly to the ADAPT model and can mostly be assigned to its basic pillars (1) Security; (2) Bonds and Networks; (3) Justice; (4) Roles and Identities; and (5) Existential Meaning [7]. Thus, it can be concluded that ADAPT is an adequate model for describing the factors that influence the mental health of refugees. Furthermore, it allowed us to order the listed super-ordinate themes and show the in-depth relationships between them, providing insights into the complex experiences of the respondents [8]. At the same time, the data analysis showed that the ADAPT model was insufficient for capturing the full range of experiences described by the ICORN respondents. Many themes could not be clearly attributed to the five basic ADAPT pillars. Therefore, it was necessary to propose two additional pillars, (6) Art and (7) Body and Mind, which would allow for an adequate reflection of the artists’ experiences. This solution resonated with the assumptions of the model, which is heuristic and open to further modifications and additions [7]. Below, the findings of this study are synthesized in the context of the current literature and our own reflections and are presented according to the super-ordinate themes mentioned above and embedded into the relevant ADAPT pillars.

### 4.1. Pillar (2): Bonds and Networks

The pillar that occurred most frequently both in the narratives of the artists interviewed and in the research findings involving other migrants/refugees [38,56,57,58,59] was Bonds and Networks. The corresponding super-ordinate theme presented within this article is that of Community.

### 4.2. Community

The theme of Community was shared by both ICORN artists and many other migrant/refugee research groups [56,57,58,59,60,61,62]. The constituent emergent themes of Community Back Home, Host Community, Family, Friends, Other Artists/Activists, Reference to Important Historical/Sociopolitical/Social/Cultural Events/Persons, Differences/Similarities between Home and Host/Other Country and Others also occurred in research involving other migrants/refugees. Common experiences connected with their countries of origin include their complex geopolitical situation and the powerlessness associated with it [61,62], revolutions, violence, and discrimination [57,58,59,61], as well as an ongoing desire to return [60] and a lack of hope for change for the better [56,58]. The joint reflections of the interviewed artists, as well as other migrants/refugees in the area of Host Community, primarily included the challenges of adapting to new everyday life [63], as well as the perceptions of these communities as safe, positive, and helpful [10], and at the same time negative, with instances of discrimination and humiliation toward refugees/migrants [58,59]. The respondents were aware that the social systems in their host countries differed from those in their countries of origin [63] and that adapting to these systems can be challenging [64]. At the same time, they tried to interact with local communities through their work [65] and build their socio-professional position from scratch [62]. It appears that both ICORN artists and other migrant/refugee respondents struggled with a lack of social networks and experienced difficulties in integrating into their host countries [24]. At the same time, the ICORN respondents felt that their positions would improve over time. A shared experience among both the ICORN artists and other migrant/refugee respondents was the sense of living in limbo between two worlds and not being fully integrated into their host communities [38,58,64]. A super-ordinate theme that occurred frequently in the narratives of the artists and other migrants/refugees was that of Family. The respondents talked about both the care and responsibility for their families [52], as well as the support and pressure experienced as a result of it [10,24]. The theme of Friends also emerged in research with ICORN artists and other migrant/refugee respondents. Friends were mainly perceived as positive and supportive [24]; however, respondents reported that these communities of friends existed in their countries of origin and that they had few or no friends in their host countries [66]. A unique experience of the ICORN artists in the context of the Friends theme was their rejection by this group at the time of migration. A theme that only occurred in the narratives of the ICORN artists was the Other Artists/Activists community and the positive interactions with it in their countries of origin. Another theme shared by the ICORN respondents and other refugee/migrant respondents was that of Reference to Important Historical/Sociopolitical/Cultural Events/Persons, where they focused primarily on describing past and current events in their countries of origin [58]. The narratives of the ICORN respondents could be distinguished by the fact that they also included references to events and figures in their host countries. Another thematic area found in the research of both ICORN artists and other migrants/refugees was that of Differences/Similarities between Home and Host/Other Country. The artists talked about the cultural similarities between the different communities [67] as well as the ostracism and marginalization experienced within them [68]. The respondents also mentioned that their everyday lives, including their financial situations, had deteriorated significantly in their host countries [56,58,69]. However, an aspect that did improve was the feeling of freedom and security [10]. What distinguished ICORN artists from other refugee/migrant artists was the promotion of social best practices from their host countries in their countries of origin. The final thematic area that was found only in ICORN artists’ narratives was that of Others, where they described other non-artistic people and communities they encountered throughout their lives.

### 4.3. Pillar (6): Art

One of the two additional ADAPT pillars proposed as a result of this study is that of Art, which was specific to the ICORN group of artists and typically does not occur in the findings of other studies involving refugees/migrants. The only references to this area can be found in the fields of literary studies and art history [67,68,70,71,72]. The super-ordinate themes assigned to this pillar and discussed within this article are Artistic Activity, Art and Migration, and Art and Persecution.

### 4.4. Artistic Activity

Within the additional pillar, Art, the key super-ordinate theme was Artistic Activity, a theme that occurred almost exclusively in the findings of this research. The ICORN respondents primarily focused on the area of Artistic/Activist Path, where they talked about their career paths, their determinants, and the most relevant interactions and collaborations. Another theme that was important for the respondents was that of Audiences, where they described their past and present audiences both in their countries of origin and in their host countries. A common issue for ICORN artists, as well as other migrant artists, is a sense of a lack of understanding by audiences in transit/host countries [72]. ICORN respondents also talked about the loss of their audiences as a result of migration and the need to adapt to new cultural rules in their host countries. Another important theme addressed by ICORN artists was that of Genres of Art Practiced, where respondents described how they worked at the intersection of different artistic genres and combined them or focused on just one area. A theme that emerged in the narratives of both ICORN respondents and other migrant/refugee artists was that of Current Artistic Activity/Activism. In their host countries, respondents had to build their professional positions from scratch, and some tried to combine this with further action for the benefit of the community in their countries of origin. A similar strategy was applied by migrant/refugee artists outside the ICORN network [65,67]. Therefore, art could be perceived as a universal bridging factor between refugees and host populations, thereby contributing to refugees’ adaptation to life in the host society. Refugees often do not share a common verbal language with the host society, but their art could be instrumental in expressing their past traumas and offering the host society insights into their potential [38,39,40]. Another issue raised by ICORN artists was that of Being a Famous Artist/Activist/Publisher/Translator. The respondents stated that with migration, they had lost the fame and recognition they had in their countries of origin, whereas other migrant/refugee artists from outside the ICORN network declared that it was only through emigration that they were recognized and became famous [71]. The final emergent theme within this super-ordinate theme was that of Inspiration/Motivation. For both ICORN respondents and other migrant/refugee artists, inspiration came not only from the people they encountered on their life journey [65] but also from their own internal conflicts, as well as their experiences and challenges in their communities in their countries of origin [68].

### 4.5. Art and Migration

Another super-ordinate theme belonging to the additional pillar, Art, is Art and Migration. This theme occurred in both the narratives of ICORN artists and research on other migrants/refugees [58,61,68,71]. In this area, the respondents focused primarily on the theme of Migration and Being an Artist, where they recounted that they never planned to migrate but that fear for their safety and that of their loved ones, the desire to continue their artistic and activist work, and the deteriorating situation in their countries of origin forced them to do so. Similar reasons for migration have also been mentioned by other non-artistic migrants/refugees [38,58]. In contrast, themes that were only addressed by ICORN artists included difficulties integrating into the artistic community in the host country, an inability to earn a living in the host country from their artistic work, and the modification of their art form and content under the influence of migration. The second area belonging to the super-ordinate theme of Art and Migration is the Choice of Migration, which distinguished ICORN artists from other migrants/refugees surveyed, as most of the network’s grantees stated that migration was not a voluntary choice. For those who perceived it as a choice, however, it was motivated solely by security issues, which has also been confirmed by the results of other studies involving refugee artists [68]. Another theme specific to the group of ICORN artists is Stopping Creation in Exile, where they shared the reasons they stopped creating art, including a lack of time due to new responsibilities and psychological problems. A common theme for both ICORN respondents and other migrant/refugee artists interviewed was that of Continuing Creation in Exile. The artists talked about the fact that they often used the existing themes of their work, that creativity was a kind of compulsion for them, and that they could easily fall into the trap of being an eternal migrant artist [71]. The final area addressed within this super-ordinate theme of Art and Migration is that of the Disadvantages of Migrating as an Artist. This is a theme mentioned by both ICORN respondents and other migrant/refugee respondents, where they shared experiences of multiple migration traumas [61] and the loss of access to a living mother tongue and their community in their countries of origin [68], as well as a lack of opportunities to publish their work [72].

### 4.6. Art and Persecution

Last but not least is the super-ordinate theme belonging to the additional pillar of Art, which was present in the narratives of ICORN artists and other migrants/refugees, which is that of Art and Persecution [57,58,65,70,73,74]. The respondents focused primarily on Persecution in the Home Country, recounting various forms of persecution in their countries of origin, including imprisonment [57,58] and surveillance [65]. What is distinctive about the ICORN artists is that the reason for their persecution usually involved their artistic work and, as a result, persecution also sometimes took other forms such as publication bans, falsifying evidence, physical violence, and attempted murder. Protection Strategies was another theme raised by both ICORN grantees and other migrants/refugees interviewed. The strategies most commonly mentioned by the respondents were fleeing and hiding [74], changing jobs and housing [73], and the use of both safe instant messaging tools and transparency by ICORN artists. An important area in the context of this super-ordinate theme is that of the Story of Persecution, where both ICORN artists and other migrant/refugee artists identified their artistic activity/activism as the beginning of their story of persecution [70]. The ICORN artists also talked a lot about the socio-political contexts of persecution and the continuation of this persecution in their host countries, which was not mentioned by other respondents. Another emergent theme that occurred in the narratives of the ICORN artists and other migrants/refugees was the Possibility/Impossibility of Going Back to the Home Country. Many of the respondents were not able to return to their countries of origin at any given time, and although they would like to [56], only a significant change in the political system would enable them to do so [72]. The ICORN artists mentioned another obstacle to returning, namely the extent to which their families had integrated into the host communities. Last but not least is the theme of Censorship/Self-Censorship within Art and Persecution, which only occurred in the statements of the ICORN respondents. Within this theme, the respondents shared both the experience of being censored by institutional factors in their countries of origin, being excluded by the communities in their host countries, and practising self-censorship.

### 4.7. Pillar (7): Body and Mind

The second additional pillar of ADAPT that was proposed as a result of this study is that of Body and Mind. It is consistent with the new ‘Five Transformations’ model proposed by Anczyk and Grzymała-Moszczyńska [4], which portrays migration as a process of change, i.e., in the area of mind and body. Themes that comprise the Body and Mind pillar can also be found in other research involving migrants/refugees [4,9,11,22,38,56,57,58,62,74]; therefore, including this area in various migrant/refugee assessment frameworks should be considered. The super-ordinate theme identified within this pillar and discussed in this article is that of Mind.

### 4.8. Mind

The theme of Mind is a super-ordinate theme that was frequently raised by both ICORN artists and other migrants/refugees [38,57,58,62,73,74]. A key issue raised in this context was Emotions as an Artistic Migrant. Both ICORN and non-ICORN artists and activists shared their primarily negative emotions about migrating as artists/activists, their difficulties with artistic/activist interactions [73], their sense of rejection by local communities, and the sadness associated with this. In addition, they also felt permanently depressed and felt there was a lack of connection with local arts/activist communities [73]. Another theme raised by respondents from ICORN and beyond was that of General Emotions in Migration. Respondents shared the diverse emotions they experienced in relation to the migration process. On the one hand, these included positive emotions such as a sense of security [62], but on the other hand, respondents also mentioned more complicated feelings such as depression [38,57] or homesickness [58,74]. Another emotion that seemed to be shared by all respondents and did not dissipate even after living in their host countries was fear [11,22,74]. The final theme that comprises the super-ordinate theme of Mind is Emotions during Persecution, where respondents shared emotions such as terror, indifference, and stress, and also a sense of tenacity.

## 5. Conclusions

In summary, the mental health challenges faced by the ICORN refugee artists were identified in the IPA analysis and presented in the form of super-ordinate themes, as discussed above, which related directly to the ADAPT model. These findings confirm that this model is adequate for systematizing and depicting in detail the experiences of migrants/refugees. At the same time, the results of this study also show that a further modification of the model is necessary so that it can fully reflect the experiences of the artists. Particular attention should be paid to the additional pillar of Body and Mind, which has the potential to become a separate category in other migrants’/refugees’ assessment frameworks. Moreover, art could be considered an important element of refugee adaptation due to its potential to bridge the gap between migrants/refugees and host societies and its ability to acknowledge not only the traumas experienced by migrants/refugees but also their capacity to contribute to host societies.

## Figures and Tables

**Table 1:** Most frequently occurring super-ordinate themes and their emerging themes in artists’ narratives.

		Theme Frequency
Bonds and Networks		
1	COMMUNITY	976
	Community Back Home	
	Host Community	
	Family	
	Friends	
	Other Artists/activists	
	Reference to Important Hist./Socio-political/Social/Cultural Events/Persons	
	Differences/Similarities between Home and Host/Other Country	
	Others	
**Art**		
2	ARTISTIC ACTIVITY	373
	Artistic/Activist Path	
	Genres of Art Practiced	
	Audiences	
	Current Artistic Activity/Activism	
	Being a Famous Artist/Activist/Publisher/Translator	
	Inspiration/Motivation	
3	ART AND MIGRATION	246
	Migration and Being an Artist	
	Choice of Migration	
	Stopping Creation in Exile	
	Continuing Creation in Exile	
	Disadvantages of Migrating as an Artist	
4	ART AND PERSECUTION	214
	Persecution in Home Country	
	Protection Strategies	
	Story of Persecution	
	Possibility/Impossibility of Going Back to Home Country	
	Censorship/Self-Censorship	
**Body and Mind**		
5	MIND	241
	Emotions of Artistic Migrants	
	General Emotions in Migration	
	Fear	
	Emotions during Persecution	

**Table 2:** Super-ordinate theme Community and its emergent themes.

Bonds and Networks			
1	COMMUNITY		
		No. of Respondents Referring to the Theme	Theme Frequency
	Community Back Home	18	324
	Host Community	18	312
	Family	15	81
	Friends	14	73
	Other Artists/activists	13	70
	Reference to Important Hist. /Socio-political/Social/Cultural Events/Persons	16	50
	Differences/Similarities between Home and Host/Other Country	16	49
	Others	6	17

**Table 3:** Super-ordinate theme Artistic Activity and its emergent themes.

Art			
2	ARTISTIC ACTIVITY		
		No. of Respondents Referring to the Theme	Theme Frequency
	Artistic/Activist Path	18	140
	Genres of Art Practiced	18	68
	Audiences	14	63
	Current Artistic Activity/Activism	15	57
	Being a Famous Artist/Activist/Publisher/Translator	13	27
	Inspiration/Motivation	7	18

**Table 4:** Super-ordinate theme Art and Migration and its emergent themes.

Art			
3	ART AND MIGRATION		
		No. of Respondents Referring to the Theme	Theme Frequency
	Migration and Being an Artist	11	103
	Choice of Migration	13	48
	Stopping Creation in Exile	7	37
	Continuing Creation in Exile	7	33
	Disadvantages of Migrating as an Artist	7	25

**Table 5:** Super-ordinate theme Art and Persecution and its emergent themes.

Art			
4	ART AND PERSECUTION		
		No. of Respondents Referring to the Theme	Theme Frequency
	Persecution in Home Country	14	94
	Protection Strategies	10	40
	Story of Persecution	13	39
	Possibility/Impossibility of Going Back to Home Country	10	25
	Censorship/Self-Censorship	9	16

**Table 6:** Super-ordinate theme Mind and its emergent themes.

Body and Mind			
5	MIND		
		No. of Respondents Referring to the Theme	Theme Frequency
	Emotions of Artistic Migrants	18	12
	General Emotions in Migration	10	68
	Fear	11	32
	Emotions during Persecution	6	31

## Data Availability

Not applicable.
